# The role of BMI, serum lipid profile molecules and their derivative indexes in colorectal polyps

**DOI:** 10.1515/almed-2023-0170

**Published:** 2024-04-26

**Authors:** Chunyu Huang, Weipeng Liang, Yuying Sun

**Affiliations:** 71067 State Key Laboratory of Oncology in South China, Guangdong Provincial Clinical Research Center for Cancer, Sun Yat-sen University Cancer Center, Guangzhou, P.R. China; Department of Endoscopy, 71067 Sun Yat-sen University Cancer Center, Guangzhou, P.R. China; 71067 Cancer Prevention Center, Sun Yat-sen University Cancer Center, Guangzhou, P.R. China

**Keywords:** body mass index, colorectal polyp, serum lipids

## Abstract

**Objectives:**

To investigate the role of body mass index (BMI), serum lipid profile molecules and their derivative indexes in colorectal polyps.

**Methods:**

A total of 352 individuals who underwent colonoscopy at our center were included in this retrospective analysis. Of these, 247 patients without evident abnormalities (control group), while 105 patients diagnosed with colorectal polyps (patient group). Serum lipid profile molecules and their derivative indexes were then compared between the two groups.

**Results:**

The patient group exhibited significantly higher levels of total cholesterol (TC) and apolipoprotein B (ApoB) compared to the control group (p<0.05). In males, the patient group displayed elevated levels of ApoB and ApoB/ApoA1 ratio compared to the control group (p<0.05). Additionally, the triglycerides (TG) and TG/high-density lipoprotein-cholesterol (HDL-C) ratios were significantly higher in the multiple polyps group than in the single polyp group (p<0.05). Furthermore, the HDL-C and HDL-C/ApoA1 ratio levels were higher in the adenomatous polyp group when compared to the non-adenomatous polyp group (p<0.05). Multiple logistic regression analysis indicated that total cholesterol (TC), TG, low-density lipoprotein-cholesterol (LDL-C), TC/HDL-C ratio, TG/HDL-C ratio and LDL-C/HDL-C ratio were risk factors for the occurrence of colorectal polyps (p<0.05). ROC curve analyses revealed that TC, ApoB, and ApoB/ApoA1 ratio were associated with colorectal polyps. No significant difference in BMI between the two groups (p>0.05).

**Conclusions:**

The incidence and progression of colorectal polyps are linked to serum lipid molecules and their derivative indexes. Dyslipidemia may increase the risk of colorectal polyps, potentially leading to colorectal cancer (CRC).

## Introduction

Colorectal polyps refer to the growth of excessive hyperplasia of the mucosa in the colon and rectum. Due to the increase in colonoscopy screenings and people’s changing lifestyle habits, the detection rate of colorectal polyps has significantly increased. Colorectal adenoma is considered a precancerous lesion since the recognized cancerous progression pattern of colorectal cancer is “adenoma-atypical hyperplasia-carcinoma” [1, 2]. Studies have shown that early removal of colorectal polyps can reduce the mortality rate of colorectal cancer [3]. Hence, exploring the risk factors associated with colorectal polyps is of great clinical significance in the prevention of colorectal cancer (CRC). However, there are controversy on the association between colorectal polyps and serum lipid levels. Studies have been shown that metabolic syndrome is closely related to the occurrence of colorectal polyps, and dyslipidemia has been identified as one of the pathogenesis factors of CRC [4], [5], [6], but other studies showed no relationship between serum lipid and colorectal polyps [7, 8]. In this study, we aim to retrospectively analyze the levels of serum lipid profile molecules and their derivative indexes in the patients with colorectal polyps and controls, observe the correlation between individual lipid levels and colorectal polyps, to explore the risk factors for the development of colorectal polyps and to provide a theoretical reference basis for the screening of high-risk groups for colorectal polyps. It can also reduce the incidence of CRC and improve people’s quality of life. This study discovered that serum lipid levels are related to colorectal polyps. Dyslipidemia, such as higher leves of total cholesterol (TC), triglycerides (TG), low-density lipoprotein-cholesterol (LDL-C), and apolipoprotein B (ApoB), may enhance the likelihood of colorectal polyps formation. This study also analyzed the relationship between lipid derivative indexes and colorectal polyps, and found that the serum lipid derivative indexes, including TC/high-density lipoprotein-cholesterol (HDL-C) ratio, TG/HDL-C ratio, LDL-C/HDL-C ratio and ApoB/apolipoprotein A1(ApoA1) ratio were related to colorectal polyps. The derivative indexes of serum lipid profile components may have changed while they are normal. The serum lipid derivative indexes, rather than the serum lipid profile molecules, are superior predictors of disease state. The results of lipid profile molecules and their derivative indexes can be obtained through simple inspection methods, which are convenient, fast, easy to repeat, of low price, and of great significance to the prevention of CRC.

## Materials and methods

### Patient population

From January 2019 to December 2020, 435 patients who had a colonoscopy at Cancer Prevention Center, Sun Yat-sen University Cancer Center, and six blood lipid tests were performed simultaneously. Most of these patients voluntarily came to our physical examination centre for a colonoscopy, and the majority of them did not consider having a disease prior to the colonoscopy, only for a regular health physical. However, 83 patients were excluded due to colon inflammation or colonic diverticulum or intestine black disease or lymphatic follicular hyperplasia, or submucosal bulge or early colon cancer and so on, resulting in a total of 352 patients enrolled in the study. Among these 352 patients, 247 were confirmed to have no polyps upon colonoscopy and were included in the control group. The remaining 105 patients were diagnosed with colorectal polyps and were assigned to the patient group. Subgroups of the patient group were created based on the colonoscopy results of the diameter, quantity, location, and tissue type of colorectal polyps. Two groups’ serum lipid profile molecules and their derivative indices were extensively examined. The patient group was further separated into different subgroups to examine the blood lipid profile molecules based on the varied clinical features of colorectal polyps. All participants involved in the study signed an informed consent form. This research was approved by the Ethics Committee of the Sun Yat-sen University Cancer Center, and followed the guidelines outlined in the Declaration of Helsinki.

Clinical data of the patients were retrospectively analyzed, including gender, age, weight, height, colorectal polyps properties (the diameter, quantity, location, and tissue type of colorectal polyps), serum lipid profile molecules and their derivative indexes. The individual serum lipid profile molecules include TG, TC, HDL-C, LDL-C, ApoA1 and ApoB, and their derivative indexes including LDL-C/HDL-C ratio, TC/HDL-C ratio, TG/HDL-C ratio, HDL-C/ApoA1ratio and ApoB/ApoA1 ratio. The diameter, quantity, location, and tissue type of colorectal polyps are all characteristics.

Patients with exclusion criteria listed as follows were excluded: (1) a surgery history of colorectal polyps and colorectal cancer; (2) other colorectal diseases apart from colorectal polyps, such as colitis, inflammatory colorectal disease, colon disease, colon diverticular, and colorectal cancer; (3) primary lipid metabolism-related diseases with intake of lipid-regulating drugs and other drugs that might affect serum lipid profile molecules in the past 3 months; (4) serious cardiac and brain diseases, as well as liver and kidney dysfunction.

### Colonoscopy

Colonoscopy using OLYMPUS CF-H290i electronic colonoscopy was performed by experienced endoscopists. All the patient group were undergone pathological biopsy, otherwise, their polyp was removed and sent for pathological examination. Two senior pathologists read the histology sections.

Patient group was further divided into <1 cm and ≥1 cm according to diameter; single and multiple according to number. It is also divided by location in the colon, with the left hemicolon consisting of the splenic flexure, descending colon, and sigmoid colon, and the right hemicolon consisting of the cecum, ascending colon, hepatic flexure, and transverse colon. According to histopathological types, they were divided into adenomatous polyps (tubular adenoma, villous adenoma, tubular-villous adenoma and serrated adenoma) and non-adenomatous polyps (inflammatory polyps, hyperplastic polyps).

### Serum lipid testing

Blood samples were collected from all participants after 8–12 h of fasting, through the cubital vein, with a volume of 3 mL. Once the plasma was separated, the levels of TC, TG, HDL-C, LDL-C, ApoA1 and ApoB were analyzed using an automatic biochemical analyzer (Cobas 8000 c702, Roche Diagnostics GmbH, Mannheim, Germany). The reference ranges of serum lipid profile molecules are: TC: 3.1–5.69 mmol/L; TG: 0.2–1.7 mmol/L; HDL-C: 1.16–1.42 mmol/L; LDL-C:2.2–3.1 mmol/L; ApoA1: 1.2–1.6 g/L; and ApoB: 0.6–1.0 g/L. According to the results of the serum lipid profile molecules, their derivative indexes include LDL-C/HDL-C ratio, TC/HDL-C ratio, TG/HDL-C ratio, HDL-C/ApoA1 ratio, and ApoB/ApoA1 ratio were calculated.

### Statistical analysis

Data analysis was performed using SPSS 25.0 statistical software (SPSS Inc. Chicago, IL, USA). Prior to conducting the t-test, a normality study was performed to assess the distribution of the data. The enumeration data were calculated as mean ± standard deviation ( $\bar{x}\pm s$ ). The comparison between different groups was analyzed using the independent sample t-test, assuming normal distribution of the data. The independent risk factors of colorectal polyps were analyzed by multiple logistic regression analysis method and ROC curve analysis. A p-value <0.05 was considered statistically significant.

## Results

### Comparative analysis of serum lipid profile molecules

There were no differences in age or gender between the control and patient groups. There were 55 males and 50 females in the patient group, with an average age of 51.14 ± 9.01. In the control group, there were 111 males and 136 females, with an average age of 49.74 ± 7.10. The levels of TC and ApoB in the patient group were significantly higher than those in the control group (p<0.05). The difference in serum lipid derivative indexes, the ApoB/ApoA1 ratio, between the two groups was statistically significant (p=0.002) (Table 1). In the patient group, ApoB (p=0.025) and ApoB/ApoA1 ratio (p=0.024) were significantly greater than in the control group among males. However, there were no significant differences among females (p>0.05) (Table 3). There was no association between body mass index (BMI) and colorectal polyps (p>0.05) (Table 1).

**Table 1:** Univariate analysis of patient group and control group.

Parameters	Patient group (n=105)	Control group (n=247)	T-value	p-Value
Gender (male, %)	55 (52.4)	111 (44.9)	1.279	0.202
Age ( $\bar{x}\pm s$ , year)	51.14 ± 9.01	49.74 ± 7.10	1.563	0.119
BMI ( $\bar{x}\pm s$ , kg/m^2^)	23.98 ± 2.62	23.39 ± 2.73	1.875	0.062
TC ( $\bar{x}\pm s$ , mmol/L)	5.75 ± 0.98	5.51 ± 0.97	2.159	0.032^a^
TG ( $\bar{x}\pm s$ , mmol/L)	1.60 ± 0.98	1.53 ± 2.35	0.314	0.754
HDL-C ( $\bar{x}\pm s$ , mmol/L)	1.46 ± 0.41	1.49 ± 0.44	−0.508	0.612
LDL-C ( $\bar{x}\pm s$ , mmol/L)	3.49 ± 0.84	3.44 ± 0.84	0.581	0.562
ApoAl ( $\bar{x}\pm s$ , mmol/L)	1.54 ± 0.25	1.58 ± 0.27	−1.216	0.225
Apo-B ( $\bar{x}\pm s$ , mmol/L)	1.10 ± 0.24	1.02 ± 0.21	3.144	0.002^a^
TC/HDL-C ( $\bar{x}\pm s$ )	4.20 ± 1.23	3.94 ± 1.20	1.800	0.073
TG/HDL-C ( $\bar{x}\pm s$ )	1.30 ± 1.14	1.23 ± 2.58	0.285	0.776
LDL-C/HDL-C ( $\bar{x}\pm s$ )	2.56 ± 0.87	2.46 ± 0.81	1.032	0.303
HDL-C/ApoAl ( $\bar{x}\pm s$ )	0.94 ± 0.13	0.93 ± 0.14	0.304	0.761
Apo-B/ApoAl ( $\bar{x}\pm s$ )	0.74 ± 0.21	0.67 ± 0.19	3.111	0.002^a^

ApoA1, apolipoprotein A1; ApoB, apolipoprotein B; BMI, body mass index; HDL-C, high-density lipoprotein-cholesterol; LDL-C, low-density lipoprotein-cholesterol; TC, total cholesterol; TG, triglycerides. ^a^p<0.05.

### The relationship between different clinical characteristics of colorectal polyps and serum lipid profile molecules

There were 105 cases in the patient group, including 30 cases of ≥45 years old, and 75 cases of <45 years old; 58 cases with single polyps, and 47 cases of multiple polyps; 18 cases with polyps ≥1 cm, and 87 cases with polyps <1 cm; 48 cases with polyps located in the right colon, and 57 located in the left colon or rectum; 64 cases with adenomatous polyps, and 41 non-adenomatous polyps (Table 2). In the patient group, ApoB and ApoB/ApoA1 ratio were significantly greater than the control group in males (p<0.05) (Supplementary Table 1), while there have no significant difference in females (p>0.05) (Supplementary Table 2).The levels of TG, TG/HDL-C ratio in the multiple polyps subgroup were significantly higher than those in the single polyp subgroup (p<0.05) (Table 3). The levels of HDL-C and HDL-C/ApoA1 ratio in the adenomatous polyps were higher than those with non-adenomatous polyps (p<0.05) (Table 4). While lipid levels in the patient group showed no correlation with age, polyp size, and polyp location (p>0.05). In general, age, locations and sizes of the polyps were not significantly correlated with the occurrence of dyslipidemia.

**Table 2:** Characteristics of the subjects.

Characteristics	n (%)
Patient group	105 (100.0)

Sex	
Males	55 (52.4)
Females	50 (47.6)
Age, years	
≤45	30 (28.6)
>45	75 (71.4)
BMI	
Normal	52 (49.5)
Overweight	47 (44.8)
Obesity	6 (5.7)
Polyps location	
Left hemicolon/rectum	57 (54.3)
Right hemicolon	48 (45.7)
Polyps size, mm	
≥10	18 (17.1)
<10	87 (82.9)
Polyps pathology	
adenoma	64 (61.0)
Non-adenoma	41 (39.0)
Polyps number	
Single	58 (55.2)
Multiple	47 (44.8)

Control group	247 (100.0)

Sex	
Males	111 (44.9)
Females	136 (55.1)
Age, years	
≤45	47 (19.0)
＞45	200 (81.0)
BMI	
Normal	153 (61.9)
Overweight	80 (32.4)
Obesity	14 (5.7)

**Table 3:** Univariate analysis of subgroup according to the number of the polyps in patient group.

Parameters	Multiple polyps group (n=47)	Single polyp group (n=58)	T-value	p-Value
TC, mmol/L	5.67 ± 0.95	5.82 ± 0.99	−0.759	0.450
TG, mmol/L	1.88 ± 1.24	1.38 ± 0.62	2.670	0.009^a^
HDL-C, mmol/L	1.39 ± 0.43	1.52 ± 0.38	−1.654	0.101
LDL-C, mmol/L	3.41 ± 0.84	3.56 ± 0.84	−0.914	0.363
ApoA1, g/L	1.50 ± 0.27	1.57 ± 0.23	−1.450	0.150
ApoB, g/L	1.11 ± 0.26	1.10 ± 0.23	0.061	0.951
TC/HDL-C ratio	4.45 ± 1.48	3.99 ± 0.96	1.894	0.061
TG/HDL-C ratio	1.65 ± 1.47	1.03 ± 0.68	2.851	0.005^a^
LDL-C/HDL-C ratio	2.69 ± 0.98	2.46 ± 0.76	1.320	0.190
HDL-C/ApoA1 ratio	0.91 ± 0.14	0.96 ± 0.13	−1.808	0.074
ApoB/ApoA1 ratio	0.77 ± 0.24	0.72 ± 0.19	1.190	0.237

ApoA1, apolipoprotein A1; ApoB, apolipoprotein B, HDL-C, high-density lipoprotein-cholesterol; LDL-C, low-density lipoprotein-cholesterol; TC, total cholesterol; TG, triglycerides. ^a^p<0.05.

**Table 4:** Univariate analysis of pathological type subgroup within the patient group.

Parameters	Adenomatous polyps (n=64)	Non-adenomatous polyps (n=41)	T-value	p-Value
TC, mmol/L	5.75 ± 0.96	5.76 ± 1.01	−0.098	0.922
TG, mmol/L	1.51 ± 1.00	1.75 ± 0.92	−1.241	0.217
HDL-C, mmol/L	1.53 ± 0.42	1.36 ± 0.36	2.086	0.039
LDL-C, mmol/L	3.49 ± 0.80	3.51 ± 0.91	−0.130	0.897
ApoA1, g/L	1.56 ± 0.25	1.50 ± 0.24	1.271	0.207
ApoB, g/L	1.09 ± 0.24	1.13 ± 0.26	−0.752	0.454
TC/HDL-C ratio	4.01 ± 1.19	4.49 ± 1.26	−1.979	0.051
TG/HDL-C ratio	1.18 ± 1.16	1.49 ± 1.10	−1.357	0.178
LDL-C/HDL-C ratio	2.45 ± 0.81	2.74 ± 0.94	−1.706	0.091
HDL-C/ApoAl ratio	0.96 ± 0.14	0.90 ± 0.11	2.617	0.010^a^
ApoB/ApoAl ratio	0.72 ± 0.21	0.77 ± 0.22	−1.276	0.205

ApoA1, apolipoprotein A1; ApoB, apolipoprotein B; HDL-C, high-density lipoprotein-cholesterol; LDL-C, low-density lipoprotein-cholesterol; TC, total cholesterol; TG, triglycerides. ^a^p<0.05.

### Analysis of independent risk factors for colorectal polyps

Multiple logistic regression analysis of the serum lipid profile molecules and their derivative indexes, using the occurrence of colorectal polyps as the dependent variable, revealed that TC, TG, LDL-C, TC/HDL-C ratio, TG/HDL-C ratio and LDL-C/HDL-C ratio were risk factors for the onset of colorectal polyps (p<0.05) (Table 5). The TC, TG, and ApoB/ApoA1 ratios were also linked to colorectal polyps, according to ROC curve analyses (Table 6). We conducted ROC curve analysis for all variables, including TC, TG, LDL-C, HDL-C, ApoA1, ApoB, TC/HDL-C ratio, TG/HDL-C ratio, and ApoB/ApoA1 ratio. Our findings revealed that TC, TG, and ApoB/ApoA1 ratio displayed comparatively higher levels of sensitivity and specificity in these outcomes.

**Table 5:** Multiple logistic regression analysis of serum lipid molecules in patient group.

Parameters	B	Standard error	Wald	p-Value	OR	95 % CI
Age ( $\bar{x}\pm s$ , year)	0.033	0.017	3.620	0.057	1.033	0.999–1.068
BMI ( $\bar{x}\pm s$ , kg/m^2^)	0.078	0.055	1.992	0.158	1.081	0.970–1.205
TC, mmol/L	8.454	2.092	16.333	<0.001^a^	4693.743	77.790–2.832 × 10^5^
TG, mmol/L	−3.459	1.310	6.976	0.008^a^	0.031	0.002–0.410
HDL-C, mmol/L	−6.325	4.811	1.729	0.189	0.002	0.000–22.280
LDL-C, mmol/L	−10.286	2.479	17.219	<0.001^a^	<0.001	0.000–0.004
ApoA1, g/L	−2.479	6.258	0.157	0.692	0.084	0.000–1.778 × 10^4^
ApoB, g/L	8.320	4.633	3.225	0.073	4105.273	0.468–3.605 × 10^7^
TC/HDL-C ratio	−8.168	2.603	9.845	0.002^a^	<0.001	0.000–0.047
TG/HDL-C ratio	3.153	1.370	5.299	0.021^a^	23.405	1.598–342.882
LDL-C/HDL-C ratio	8.108	3.045	7.092	0.008^a^	3321.874	8.508–1.297 × 10^6^
HDL-C/ApoA1 ratio	0.375	11.000	0.001	0.973	1.455	0.000–3.355 × 10^8^
ApoB/ApoA1 ratio	−0.268	6.481	0.002	0.967	0.765	0.000–2.513 × 10^5^

ApoA1, apolipoprotein A1; ApoB, apolipoprotein B; B, regression coefficient; BMI, body mass index; HDL-C, high-density lipoprotein-cholesterol; LDL-C, low-density lipoprotein-cholesterol; OR, odds ratio; TC, total cholesterol; TG, triglycerides; 95 % CI=95 % confidence interval. ^a^p<0.05.

**Table 6:** ROC curve analysis results of the diagnostic value of related lipid indexes for colorectal polyps.

Parameters	AUC	SE	p-Value	95 % CI	Cut-off value	Sensitivity	Specificity	Youden index
TC	0.575	0.033	0.025^a^	0.510–0.641	6.1250	0.371	0.765	0.137
TG	0.561	0.034	0.069	0.494–0.628	1.8750	0.295	0.830	0.125
HDL-C	0.487	0.034	0.692	0.420–0.554	1.2650	0.686	0.352	0.038
LDL-C	0.508	0.034	0.812	0.442–0.574	2.6700	0.886	0.170	0.056
ApoA1	0.454	0.034	0.170	0.388–0.520	1.2050	0.962	0.069	0.031
ApoB	0.602	0.034	0.002^a^	0.535–0.669	1.2050	0.381	0.826	0.207
TC/HDL-C	0.562	0.034	0.065	0.495–0.630	5.2150	0.229	0.899	0.127
TG/HDL-C	0.548	0.034	0.152	0.481–0.615	1.5750	0.276	0.834	0.110
ApoB/ApoA1	0.598	0.034	0.003^a^	0.531–0.665	0.7650	0.495	0.717	0.212

ApoA1, apolipoprotein A1; ApoB, apolipoprotein B; HDL-C, high-density lipoprotein-cholesterol; LDL-C, low-density lipoprotein-cholesterol; TC, total cholesterol; TG, triglycerides; 95 % CI=95 % confidence interval. ^a^p<0.05.

## Discussion

CRC is one of the most common malignant tumorsglobally, with high morbidity and mortality rates [9]. CRC has a low detection rate in general population screening, and there is no easy and effective method for population census. Fecal hemoglobin detection tests, is a common non-invasive examination, which are increasingly utilized worldwide in colorectal cancer screening programs, were not performed in this study due to poor compliance among participants. Endostroscopy is the standard for the diagnosis of lesions [10], but due to the severe shortage of experienced endoscopists in China, and the fact that endoscopy is an invasive and expensive test that is difficult for many asymptomatic people to accept, it is difficult to carry out CRC screening using endoscopy, and the only potentially effective method is to screen the population at high risk of CRC. The aim of the present study is to assist in determining the population at risk of CRC by identifying high risk factors for colorectal polyps, and to act as a guide for CRC screening. Therefore, studying the risk factors of colorectal polyps is of great significance for the reduction of the incidence of CRC.

Studies have found that dyslipidemia plays a critical role in the occurrence and development of a variety of cancers, which might be a potential target for anti-cancer therapy [11]. The main characteristics of cancer tissue are that the body loses control and endures excessive cell proliferation, resulting in the formation of a cancer. Fast cell growth demands the addition of more cholesterol because cholesterol is the major component of cell membranes [12, 13]. Dyslipidemia and colorectal cancer have a strong association. Dyslipidemia was shown to be substantially more common in people with CRC. Diabetes, hyperlipidemia, and inflammatory bowel illness are all independently related to the risk of multiple CRC, according to a large retrospective case-control research in China [4]. Due to the popularity of endoscopic technology, the detection rate of colorectal polyps has increased significantly in recent years. While it is reported that colorectal polyps are associated with age, gender, smoking, drinking, high-fat diet, obesity, diabetes, metabolic syndrome and other factors, the etiology and pathogenesis of colorectal polyps are still unclear. Some studies suggest that dyslipidemia is also closely related to colorectal adenoma [14], [15], [16], [17], [18], [19], [20], [21], but there is still controversy. Some researchers believe that dyslipidemia has nothing to do with colorectal adenoma [7, 8], while others believe it is a risk factor for their occurrence and progression. Polyps and serum lipids, on the other hand, were the subject of very few studies [22], [23], [24], [25]. In colorectal polyps, we found that significantly higher levels of TC and ApoB than non-polyp (Table 1), which demonstrated that dyslipidemia possibility associated with colorectal polyp, consistent with the previous investigations.

At present, the correlation of dyslipidemia and colorectal polyps is not yet clear. Studies have found that TG can not only activate insulin-like growth factor-1 and regulate the activity of K-ras protein, but also stimulate the abnormal proliferation of intestinal epithelial cells and promote the transformation of colorectal adenoma into tumor [17]. Studies have also suggested that dyslipidemia activates the MAPK pathway by increasing the reactive oxygen species activity of colon adenoma cells, leading to excessive cell proliferation and promoting the development of polyps into tumors [26]. High levels of TG can increase pro-inflammatory factors while decreasing inflammatory inhibitory cytokines, which affects the growth, apoptosis and proliferation of tumors and stromal cells, and promotes the carcinogenesis of adenomas [27]. Dyslipidemia can also cause oxidative stress and work together with inflammation to produce carcinogenic effects. Considered as an important pathogenic factor of many diseases, oxidative stress will lead to the production of more reactive oxygen species, lipid peroxidation, lipid macromolecules of cell membrane into lipid peroxide, causing tissue damage. This demonstrates that oxidative stress and lipid peroxidation are linked, and that the degree of lipid peroxide is connected to serum cholesterol and TG [28, 29]. In addition, the increase of serum lipid profile molecules can also change the excretion and circulation of bile acid, lead to the mechanism of bile acid absorption or butyric acid inhibition, increase the content of secondary bile acid in the intestine, and cause DNA damage, cell proliferation and angiogenesis microenvironment, promotes the occurrence of colorectal polyps [20].

Studies have suggested that serum lipid derivative indexes are related to the occurrence and development of some cancers. Some studies report that the ApoB/ApoA1 ratio is associated with the increase of overall cancer incidence in males [30]. In this study, they also find results regarding ApoB, which is also associated. Furthermore, focusing on patients with CRC, they observe a relationship with ApoB, not with ApoA1, similar to the findings of this study. The ApoB/ApoA1 ratio rose in patients with gastric cancer (GC), according to Kimak et al. and Ma et al. [31, 32]. Increased preoperative serum LDL-C/HDL-C ratio is an independent risk factor for CRC tumor–node–metastasis staging and is closely related to CRC prognosis [33]. However, there are currently very few studies on the relationship between serum lipid derivative indexes and the occurrence and development of colorectal polyps. Therefore, we further analyzed the relationship between colorectal polyps and serum lipids and their derivatives, and this article is to study these correlations based on their location, size, number and pathological types. The present study found that dyslipidemia, such as TG, TC, LDL-C, ApoB, TC/HDL-C ratio, TG/HDL-C ratio, LDL-C/HDL-C ratio, and ApoB/ApoA1ratio, plays an important role in the occurrence and development of colorectal polyps. This finding aligns with studies conducted by Isakov et al., demonstrating an association between a high TG/HDL ratio and serrated polyps [34]. We further analyzed the relationship between colorectal polyps and serum lipids derivative indexes in subgroup, and found that the age, location, and size of polyps are not significantly correlated with dyslipidemia. According to multiple regression analysis, TC, TG, LDL-C, and ApoB may increase the incidence of colorectal polyps (p<0.05). The TC, ApoB and ApoB/ApoA1 ratios were also linked to colorectal polyps, according to ROC curve analyses. These results are consistent with the previous research [23].

We also studied the relationship between lipids and BMI in colorectal polyps. Previous studies have tested the lipid association with BMI, yielding inconclusive results [34], [35], [36]. In present study, we found that there is no meaningful correlation between BMI and colorectal polyps.

There were several limitations in our research. This study was conducted in a single location, and the sample size was relatively small. Because the subjects in this study were selected among people who attended to the hospital for routine health exams and colonoscopies, there could have been a selection bias. Moreover, the current research on the related risk factors of colorectal polyps are not yet perfect, and it is necessary to expand the sample size to obtain more accurate results. Thus, our results are preliminary.

In summary, this study found that dyslipidemia plays an important role in the occurrence and development of colorectal polyps, and dyslipidemia is an independent risk factor for colorectal polyps. We found that serum lipid profile molecules and their derivative indexes could be used as predict factors in the process of colorectal polyps. People with dyslipidemia need colonoscopy more than the general population.

## Supplementary Material

Supplementary Material
