# Supplementary material for: The role of BMI, serum lipid profile molecules and their derivative indexes in colorectal polyps
Source: Adv Lab Med. 2024 Apr 26;5(3):276–82. doi: 10.1515/almed-2023-0170 (PMC11381085; doi:10.1515/almed-2023-0170)
Supplement: Supplementary file 1 — Supplementary Material [file j_almed-2023-0170_suppl_001.docx]

**Supplementary Table 1.** Univariate analysis of the patient group versus the control group for males

| Parameters | Patient Group(n=55) | Control Group(n=111) | T value | *P* value |
| --- | --- | --- | --- | --- |
| Age($\bar{x}\pm s,$Year) | 49.49$\pm$9.13 | 48.59$\pm$7.77 | 0.660 | 0.510 |
| BMI($\bar{x}\pm s,$kg/m^2^) | 24.47$\pm$2.71 | 24.24$\pm$2.65 | 0.542 | 0.589 |
| TC(mmol/L) | 5.70$\pm$0.77 | 5.49$\pm$0.99 | 1.429 | 0.155 |
| TG(mmol/L) | 1.87$\pm$1.15 | 1.70$\pm$3.24 | 0.367 | 0.714 |
| HDL-C(mmol/L) | 1.30$\pm$0.31 | 1.37$\pm$0.46 | -0.970 | 0.334 |
| LDL-C(mmol/L) | 3.50$\pm$0.66 | 3.44$\pm$0.86 | 0.444 | 0.658 |
| ApoA1(g/L) | 1.45$\pm$0.22 | 1.49$\pm$0.28 | -1.039 | 0.300 |
| ApoB(g/L) | 1.14$\pm$0.20 | 1.06$\pm$0.21 | 2.265 | 0.025* |
| TC/HDL-C ratio | 4.62$\pm$1.20 | 4.28$\pm$1.26 | 1.666 | 0.098 |
| TG/HDL-C ratio | 1.65$\pm$1.37 | 1.46$\pm$3.52 | 0.378 | 0.706 |
| LDL-C/HDL-C ratio | 2.83$\pm$0.79 | 2.69$\pm$0.87 | 1.045 | 0.298 |
| HDL-C/ApoA1ratio | 0.89$\pm$0.11 | 0.90$\pm$0.12 | -0.664 | 0.508 |
| ApoB/ApoA1ratio | 0.81$\pm$0.20 | 0.73$\pm$0.19 | 2.272 | 0.024* |

Abbreviations: ApoA1=Apolipoprotein A1; ApoB=Apolipoprotein B; BMI=Body Mass Index; HDL-C=high-density lipoprotein-cholesterol; LDL-C=low-density lipoprotein-cholesterol; TC=Total cholesterol; TG=triglycerides.

*p<0.05

**Supplementary Table 2.** Univariate analysis of the patient group versus the control group for females

| Parameters | Patient Group(n=50) | Control Group(n=136) | T value | *P* value |
| --- | --- | --- | --- | --- |
| Age($\bar{x}\pm s,$Year) | 52.96$\pm$8.61 | 50.67$\pm$6.40 | 1.964 | 0.051 |
| BMI($\bar{x}\pm s,$kg/m^2^) | 23.45$\pm$2.43 | 22.71$\pm$2.61 | 1.738 | 0.084 |
| TC(mmol/L) | 5.81$\pm$1.17 | 5.53$\pm$0.96 | 1.664 | 0.098 |
| TG(mmol/L) | 1.31$\pm$0.62 | 1.39$\pm$1.20 | -0.422 | 0.674 |
| HDL-C(mmol/L) | 1.64$\pm$0.43 | 1.59$\pm$0.40 | 0.801 | 0.424 |
| LDL-C(mmol/L) | 3.49$\pm$1.00 | 3.43$\pm$0.83 | 0.366 | 0.715 |
| ApoA1(g/L) | 1.64$\pm$0.25 | 1.64$\pm$0.25 | -0.165 | 0.869 |
| ApoB(g/L) | 1.07$\pm$0.28 | 0.99$\pm$0.21 | 1.975 | 0.050 |
| TC/HDL-C ratio | 3.73$\pm$1.11 | 3.67$\pm$1.08 | 0.354 | 0.724 |
| TG/HDL-C ratio | 0.92$\pm$0.66 | 1.04$\pm$1.41 | -0.553 | 0.581 |
| LDL-C/HDL-C ratio | 2.26$\pm$0.87 | 2.28$\pm$0.72 | -0.110 | 0.912 |
| HDL-C/ApoA1ratio | 0.99$\pm$0.13 | 0.96$\pm$0.14 | 1.428 | 0.155 |
| ApoB/ApoA1ratio | 0.67$\pm$0.21 | 0.62$\pm$0.16 | 1.711 | 0.089 |

ApoA1=Apolipoprotein A1; ApoB=Apolipoprotein B; BMI=Body Mass Index; HDL-C=high-density lipoprotein-cholesterol; LDL-C=low-density lipoprotein-cholesterol; TC=Total cholesterol; TG=triglycerides.

*p<0.05
